# Quality Assessment of Deep-Frying Palm Oil by Impedimetric Sensing with a Simple and Economic Electrochemical Cell

**DOI:** 10.3390/s21217093

**Published:** 2021-10-26

**Authors:** Yi Kung, Bo-Chuan Hsieh

**Affiliations:** Department of Biomechatronics Engineering, College of Bio-Resources and Agriculture, National Taiwan University, Taipei 10617, Taiwan; d99631001@ntu.edu.tw

**Keywords:** cell constant, screen-printed carbon electrode, total polar compounds, acid value, viscosity

## Abstract

Quality control of deep-frying oil is a global public health concern. A simple and economic electrochemical chamber composed of two bare screen-printed carbon electrodes (working area: 78.54 × 10^2^ cm^2^; distance: 0.0055 cm; cell constant: 0.70 × 10^−2^ cm^−1^) was constructed for precisely acquiring the impedimetric responses of a high-resistance palm oil sample (RSD < 7%, *n* = 3). Good correlations between the measured impedance data (charge transfer resistance and logarithmic output impedance (Log Z) obtained in the frequency region <0.1 Hz) and the regulatory quality indicators (total polar compounds and acid value) were achieved (*R*^2^ > 0.97), suggesting that the proposed impedimetric sensing method is useful for accurately assessing the deteriorated condition of repeated frying oil. Applications for rapid screening can also be realized because the measurement times of Log Z at any given perturbation frequency from 0.01–1 Hz were all less than 3 min.

## 1. Introduction

Deep-frying is a popular cooking method that efficiently brings a savory flavor and crunchy surface texture to our daily foods. However, repetitive and continuous heating processes gradually deteriorate the quality of deep-frying oil via a complex series of chemical reactions (such as hydrolysis, oxidation, and polymerization). These provoke the accumulation of a confusing number of toxic and carcinogenic compounds, which may increase the risk of developing cancers, Alzheimer’s, and Parkinson’s diseases [1]. It is, therefore, of great importance to monitor the quality of deep-frying oil during repeated usage, especially for food service industries and official food control agencies.

Several physical and chemical parameters of used frying oil have been suggested as the deterioration indicators, including foam persistence, smoke point, turbidity, viscosity, acid value (AV), total polar compounds (TPC), peroxide value, iodine value, carbonyl value, and *p*-anisidine value [2,3]. Among these, TPC is the most representative and widely accepted quality indicator above which the deep-frying oil should be discarded. The maximum limit of TPC content is set between 24% and 27% according to the laws or regulations of many countries [4]. Since the main constituents of fresh edible oil are almost nonpolar triacylglycerols, it decomposes easily at high cooking temperature, which ends with numerous thermo-oxidized polar byproducts (including free fatty acids, alcohols, aldehydes, ketones, and peroxides), which are classified as the so-called TPC [5]. Although strong positive correlations between TPC content and deep-frying cycle (even with different frying media) have been extensively demonstrated [6,7,8], the standard open column chromatography for TPC determination is still too complicated, costly, and time-consuming, which is not suitable for routine usage.

A variety of alternatives have been recently proposed by examining other easily and quickly obtained parameters that are well correlated to TPC. For example, the measurement of dielectric constant showed potential in monitoring the deterioration of palm oil, where a good positive linear correlation with TPC (*R*^2^ = 0.98) was achieved over 5 days of frying [9]. A highly linear relationship between electrical conductivity and TPC (*R*^2^ = 0.9853) was also established over 40 h of nonconsecutive frying [10]. The capillary penetration rate of high-temperature-treated sunflower and virgin olive oils was proven to possess a negative linear dependency (*R*^2^ = 0.91) against TPC, and the measurement time was only a few minutes [11]. UV/Vis spectrophotometric absorbance at 490 nm was also used to examine the degradation of sunflower oil during intermittent deep-frying for 5 consecutive days, and the corresponding results exhibit a good agreement with TPC (*r* = 0.999) in the same set of oil samples [12]. The analysis of electrical capacitance of deep-frying palm oil was demonstrated using a capacitive sensor probe consisting of an interdigitated gold electrode; the highest positive correlation between TPC and capacitance measurement was found at 100 kHz with *R*^2^ of 0.90 [13]. The same experimental design was also adopted for investigating the difference in impedance value at several heating intervals, and a good correlation (*r* = 0.9847) with TPC change was achieved at lower frequency of 100 Hz [14].

The applications of electrical and electrochemical impedance spectroscopy (EIS) on food quality assessments and food safety inspections have been documented in several reviews over the past decade [15,16]. Since EIS offers multiple appealing advantages in terms of low cost, test speed, ease of operation, and simplicity of design, it has potential in developing portable devices to replace traditional laboratory apparatus. Many efforts were consequently made using commercial integrated circuit chips to realize miniaturization purposes. For example, a battery-powered impedance converter IC chip (AD5933 by Analog Devices, Norwood, MA, USA) was incorporated into an impedance spectroscopy unit for biosensor applications that showed the capability of accurately obtaining an EIS response from 10 Ω to 2 MΩ in the frequency range of 100  Hz–100  kHz [17]. Later, the same research group further extended the impedance measurable range and frequency bandwidth to 10 Ω–5 MΩ and 10  Hz–100  kHz, respectively [18]. To meet the requirements of measuring broader extent of impedance (10 Ω–10 GΩ) in a wider frequency range (0.01 Hz–100 kHz) for anticorrosion protection diagnostics, a handheld impedance module was also developed with the aid of two AD5933 chips [19]. More applications of utilizing commercially available IC chips for impedance measurements can be found elsewhere [20].

This article aims to investigate the electrochemical impedance spectroscopic behavior of palm oil during repetitive deep-frying of French fries for 24 h, whereas the relationships between impedimetric measurements and the regulatory quality indicators (AV and TPC by conventional methods) of deep-fried oil were also examined. The novelty of this work lies in the simple and economic strategy of using a pair of screen-printed carbon electrodes to optimize the geometry of the electrochemical chamber (i.e., cell constant). Theoretically, the obtained impedimetric sensing response can be effectively lowered with a decrease in cell constant; accordingly, the potential of realizing in-field quality assessment by incorporating the aforementioned portable devices is also discussed.

## 2. Materials and Methods

### 2.1. Reagents, Samples, and Electrodes

Diethyl ether, ethanol, and phenolphthalein were purchased from Sigma-Aldrich, Inc. (St. Louis, MO, USA). Potassium hydroxide was bought from Nacalai Tesque, Inc. (Kyoto, Japan). Three commercial palm oils were obtained from Master Channels Co., Ltd. (Taipei, Taiwan), Dachan Great Wall Group (Tainan, Taiwan), and Taisun Enterprise Co., Ltd. (Changhua, Taiwan). Frozen French fries were supplied by Long Feng Food Co., Ltd. (Taipei, Taiwan). Screen-printed carbon electrodes with a working area of 5 mm in diameter (CC-GRP-1), 10 mm in diameter (CC-GRP-2), 15 mm in diameter (CC-GRP-3), 20 mm in diameter (CC-GRP-4), and 25 mm in diameter (CC-GRP-5) were provided by Vida Biotechnology Co., Ltd. (Taichung, Taiwan).

### 2.2. Deep-Frying Process

At the beginning of every batch of experiment, 5 L of fresh palm oil was preheated at 180 ± 5 °C for 5 min by a deep-fat fryer (WFT-8L, Daching Food Equipment, Taipei, Taiwan). Each frying cycle was performed by introducing 250 g of French fries; one frying cycle of 15 min consisted of frying French fries for 5 min and idle heating for 10 min. A total of 96 consecutive cycles were conducted without any oil replenishment. Approximately 100 mL of oil sample was collected once every 16 frying cycles (seven samples in total, including the fresh one), filtered through Whatman No. 1 filter paper (pore size: 11 μm) to remove suspended food particles, and stored in brown bottles at 4 °C for further analysis.

### 2.3. EIS Measurement

**Figure 1:**
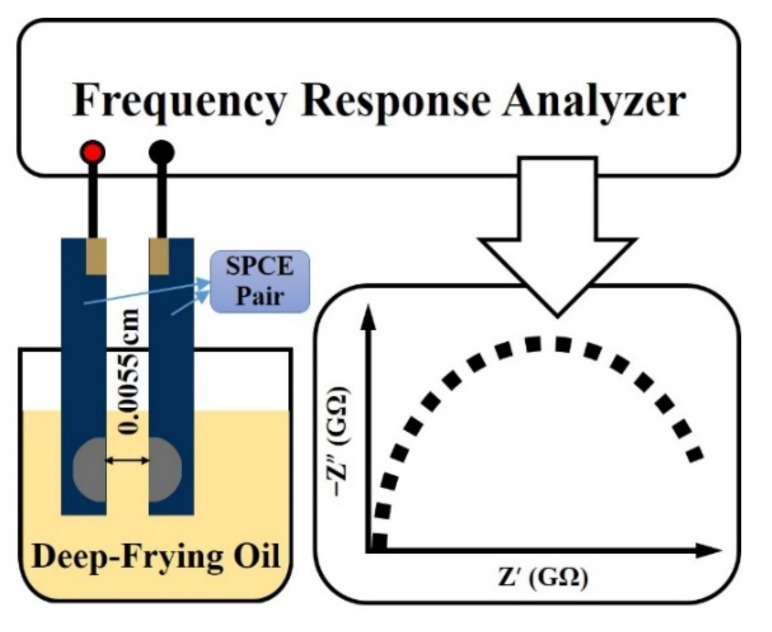
Schematic diagram of the EIS measurement setup.

As shown in Figure 1, 30 mL of frying oil sample was preheated in a beaker at 30 ± 0.5 °C for 5 min by a dry bath incubator (MD-01N, Bioman Scientific Co., Ltd., Taipei, Taiwan) and controlled at the same temperature throughout the following experiment. Two identical screen-printed carbon electrodes were paralleled face to face and separated by a polyethylene thin film (PR5#, 0.055 mm in thickness, Symbio Inc., Taipei, Taiwan) outside of the working area; this electrochemical cell was then gently immersed into the oil sample and kept for 2 min to achieve a uniform sample distribution between electrodes via the capillary effect [21]. The cell constant of an electrochemical chamber is given as k = L/A, where L is the distance between two electrodes, and A is the working area of the electrode [22]. EIS measurements were conducted using an Autolab PGSTAT-30 system equipped with an FRA2-module (Eco Chemie B. V., Utrecht, Netherlands) that scanned from 1 Hz down to 0.01 Hz with an unbiased 100 mV sinusoidal potential input. The obtained experimental spectra were interpreted using the simplified Randles equivalent circuit model, which consists of a solution resistance (R_S_) in series with a parallel combination of a double-layer capacitance (C_D_) to a charge transfer resistance (R_CT_).

### 2.4. Viscosity

Eight milliliters of each frying oil sample was poured into a small sample adaptor (SSA-18/13R, AMETEK Brookfield, MA, USA.), and the viscosity was determined by a rotational viscometer (DV2TLV, AMETEK Brookfield, Middleborough, MA, USA) with No. SC4-18 spindle at 30 ± 0.5 °C (temperature control was achieved by a circulating water jacket) at a shear rate of 30 rpm for 10 min.

### 2.5. Acid Value

Acid values (AV) of frying oils were determined according to the ISO method [23]. Ten grams of the test portion was thoroughly mixed with 150 mL of ethanol/diethyl ether (1:1, *v*/*v*) solution; 0.3 mL of phenolphthalein indicator (1% in ethanol) was then added, and gradually titrated with standard potassium hydroxide solution (0.1 N in ethanol) to a permanent pink endpoint. The acid value was calculated using the following equation:

Acid value (mg KOH/g oil) = (V × C × 56.1)/W,

where V is the volume of KOH used for titration (mL), C is the concentration of KOH (0.1 N in this study), and W is the weight of test portion (10 g in this study).

### 2.6. Total Polar Compounds

The total polar compounds (TPC) content of frying oils was determined using a deep-frying oil tester (Testo 270, Testo Inc., Lenzkirch, Germany) at 180 ± 5 °C; the sensor plate was immersed into the hot oil sample during the frying process and held for 20 s to acquire a stable reading. Before each batch of experiments, the instrument was calibrated according to the manufacturer’s instruction manual using Testo reference oil (order no. 0554 2650) for its measuring accuracy.

## 3. Results and Discussion

### 3.1. Effect of Electrochemical Cell Geometry on Impedimetric Sensing

**Figure 2:**
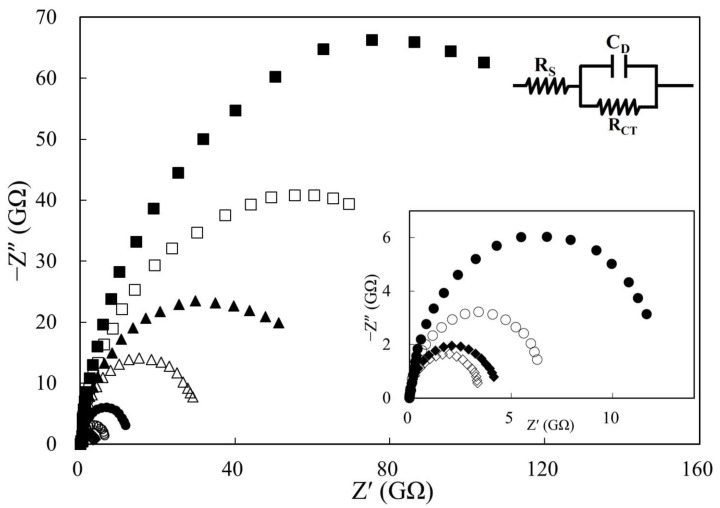
Nyquist diagrams of fresh palm oil with a cell constant of 19.61 × 10^−2^ (■), 14.01 × 10^−2^ (□), 8.40 × 10^−2^ (▲), 2.80 × 10^−2^ (Δ), 0.70 × 10^−2^ (●), 0.31 × 10^−2^ (○), 0.18 × 10^−2^ (♦), and 0.11 × 10^−2^ (♢) cm^−1^. Applied potential: 0 V; amplitude: 100 mV; frequency range: 1–0.01 Hz; temperature: 30 °C. The inset shows a closer view of the Nyquist curves with lower cell constants, as well as the equivalent circuit model.

The electrical property of edible oil is generally considered an insulator because of its low conductivity (nS·m^−1^) [24]. It is hard to obtain precise impedimetric sensing data with such a high resistance substance in the conventional three-electrode electrochemical design mainly due to the impedance mismatching problem. A two-electrode thin-layer configuration with a low cell constant was proposed to effectively lower the interfacial ohmic drop and successfully applied to measure the impedance of a high-resistance biodiesel sample [21]. The easiest way to minimize the cell constant is by either enlarging the effective surface area of the electrode or by coming as close as possible to the two electrode surfaces. As shown in Figure 2, the size of the semicircular Nyquist curve of fresh palm oil increased upon gradually raising the cell constant from 0.11 × 10^−2^ to 19.61 × 10^−2^ cm^−1^. This phenomenon implies an obstacle to generate exchange current flow at the electrode interface with high-cell-constant configurations, leading to significant rises in the charge transfer resistance (R_CT_ in Table 1). The inverse relationship between the measured double layer capacitance (C_D_ in Table 1) and cell constant is also in agreement with theoretical expectation [25]. However, a notable feature of the Nyquist plot was the lack of a diffusion-controlled component in the low-frequency region (generally a diagonal line with an slope of 45°), which may have resulted from our unique electrochemical cell design, where the two screen-printed carbon electrodes were parallelly placed at a very close distance of 0.0055 cm, which is even closer than an earlier study (L = 0.0080 cm) [21].

A simplified Randles equivalent circuit model without the Warburg diffusion element was, therefore, chosen for obtaining more accurate impedance fitting data. Although the precision of R_CT_ for three repeated measurements could be effectively improved by shortening the distance between electrode surfaces (RSD = 18.23% for L = 0.0385 cm, and RSD = 4.08% for L = 0.0055 cm), further geometric adjustment in expanding the electrode area showed an unexpected trend with a change in cell constant (RSD = 5.72% for A = 78.54 × 10^2^ cm^2^, but RSD = 27.10% for A = 490.87 × 10^2^ cm^2^). According to our observations, this may be attributed to the limitation of capillary action, where a uniform sample distribution in the electrochemical cell could only be achieved with geometries #4 and #5 (2 min of immersing time in oil sample before impedimetric measurement). Even though an additional extension of immersing time to 30 min was performed for all other geometries, no obvious improvement in sample distribution was noted. By considering both precision (lower RSD) and ease of portability (lower R_CT_ value), geometry #5 was selected as the best architecture (L = 0.0055 cm, A = 78.54 × 10^2^ cm^2^, cell constant = 0.70 × 10^−2^ cm^−1^) for the subsequent experiments.

The effect of different kinds of commercial palm oils on impedimetric sensing (conducted using geometry #5 in triplicate tests) was also examined for practical concern in the early stage. Good repeatabilities in R_CT_ measurements were found for all three brands of fresh palm oils in terms of RSD ranging from 5.10% to 6.27%.

### 3.2. Effect of Deep-Frying Cycle on Impedimetric Sensing

**Figure 3:**
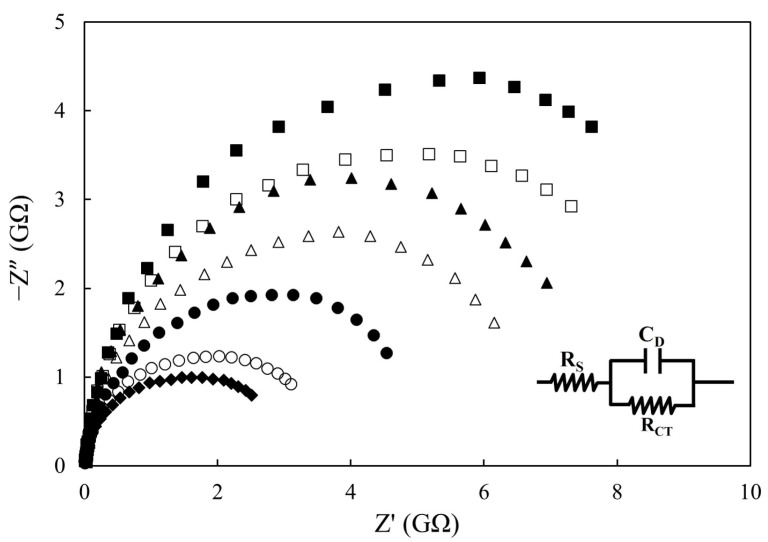
Nyquist diagrams of palm oil during deep-frying process: (■) fresh oil, (□) after 4 h, (▲) after 8 h, (Δ) after 12 h, (●) after 16 h, (○) after 20 h, and (♦) after 24 h deep-frying of French fries. The inset shows the equivalent circuit model. The configuration of the electrochemical cell was geometry #5 (refer to Table 1: electrode No. CC-GRP-2, 10 mm in diameter). Applied potential: 0 V; amplitude: 100 mV; frequency range: 1–0.01 Hz; temperature: 30 °C.

Figure 3 presents the Nyquist spectra of palm oil during deep-frying process, whereby the size of the semicircle gradually decreased with the deep-frying cycle. This phenomenon can be ascribed to the cumulative generation of both electrochemically active species and polar byproducts in the repeated cooking oil that significantly improved the interfacial electron transfer efficiency between the electrode pair. It has been proven that high temperature renders the polyunsaturated fatty acids contained in edible vegetable oils prone to producing unstable lipid oxidation products, such as hydroperoxides, aldehydes, and epoxides [26]. Among them, phospholipid hydroperoxides were reported to exhibit electrochemical redox activity even at very low overpotentials [27,28]. Referenced data also indicate that the presence of polar compounds in oil sample can lower the impedimetric sensing response due to the elevation of conductivity [29]. Consequently, an evident trend of decrease in R_CT_ values was observed (Table 2). Among the three equivalent circuit elements, only R_CT_ showed a strong linear relationship with deep-frying cycle (*R*^2^ = 0.9807), as expected.

**Figure 4:**
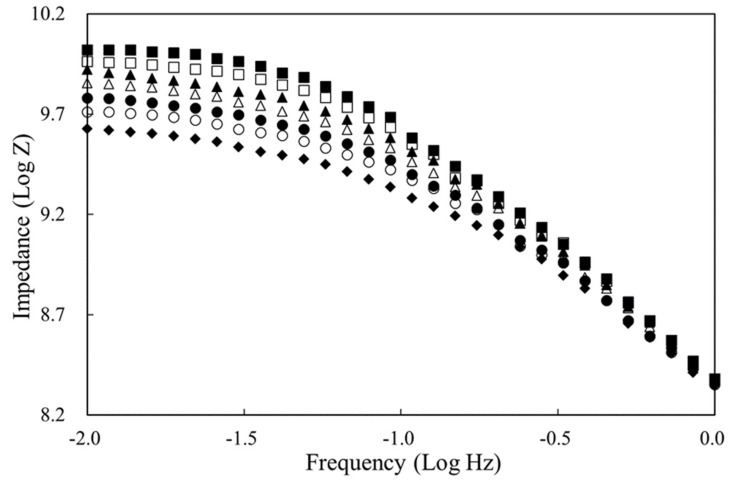
Bode plots of palm oil during deep-frying process: (■) fresh oil, (□) after 4 h, (▲) after 8 h, (Δ) after 12 h, (●) after 16 h, (○) after 20 h, and (♦) after 24 h deep-frying of French fries. The configuration of the electrochemical cell was geometry #5 (refer to Table 1: electrode No. CC-GRP-2, 10 mm in diameter). Applied potential: 0 V; amplitude: 100 mV; frequency range: 1–0.01 Hz; temperature: 30 °C.

The frequency-dependent impedimetric behaviors of oil samples are depicted in the Bode plots of Figure 4 (these data corresponded to the above Nyquist curves), whereby the magnitude of output impedance (Log Z) consistently declined upon prolonging the cooking period in the full frequency range. Satisfactory linear relationships (*R*^2^ > 0.99) between Log Z and deep-frying cycle were found at every frequency point lower than 0.1 Hz. According to our experience of conducting EIS with a high-resistance biodiesel sample, the differences in Log Z in the lower-frequency region usually exhibit better resolution for sensing purposes because of the reduced diffusion constraint for redox reactions [21]. Therefore, a relatively narrow extent of perturbation frequency was employed (0.01–1 Hz in this study) for both rapidity and precision.

### 3.3. Effect of Deep-Frying Cycle on Viscosity, AV, and TPC

The viscosity, AV, and TPC of oil samples as affected by deep-frying cycle are summarized in Table 3. The progressive increase in viscosity clearly indicated the accumulative formation of high-molecular-weight polymeric compounds via the combined result of thermal oxidation and polymerization of triacylglycerol in the frying medium. In addition, the continuous generation of viscous materials caused a tendency toward increasing foam persistence under our observation, especially after 60 repeated frying batches. AV is one of the regulatory indicators of edible oil deterioration, which mainly measures the content of free fatty acids produced during the triacylglycerol hydrolytic reaction happening within the frying operation. A consistent increase in the AV value from 0.21 to 1.91 mg KOH/g was noticed, and the final AV was very close to the limit of 2 mg KOH/g set by the Taiwan regulation [6], suggesting that the oil condition almost reached the discard point after 24 h of repeated frying with French fries. The TPC content also gradually increased as the frying time extended, reaching 26.33% at the end of the frying experiment. This TPC value exceeds the Taiwanese regulatory limit of 25% and, thus, the oil should be disposed of properly [6]. Overall, the viscosity, AV, and TPC were found to possess good linear dependencies vs. deep-frying cycle with *R*^2^ equal to 0.9914, 0.9893, and 0.9909, respectively.

### 3.4. Relationships between Impedimetric Data and Regulatory Quality Indicators

**Figure 5:**
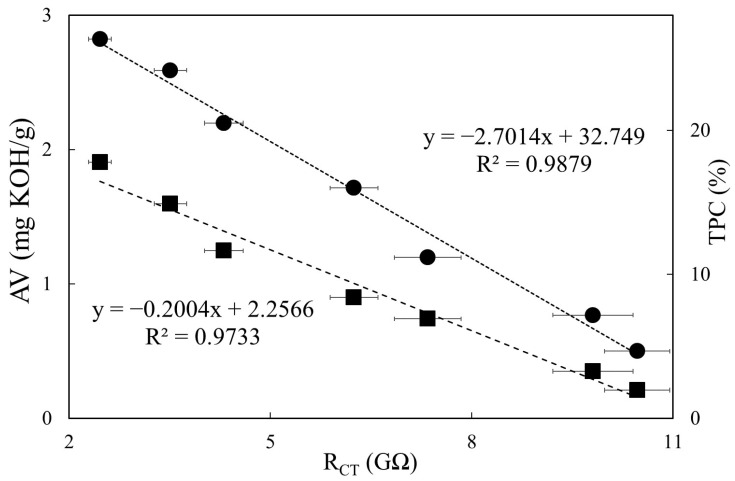
Comparisons of R_CT_ with AV (■) and TPC (●) during the 24 h deep-frying process. The R_CT_ values were from Table 2; the AV and TPC values were both from Table 3.

To understand the feasibility of assessing deep-frying oil deterioration by our impedimetric sensing approach, experimental data of R_CT_ and Log Z were used to examine their relationships vs. AV and TPC, respectively. As can be seen in Figure 5, the linear fittings with respect to R_CT_ showed significant negative correlations with *R*^2^ values of 0.9733 for AV and 0.9878 for TPC.

**Figure 6:**
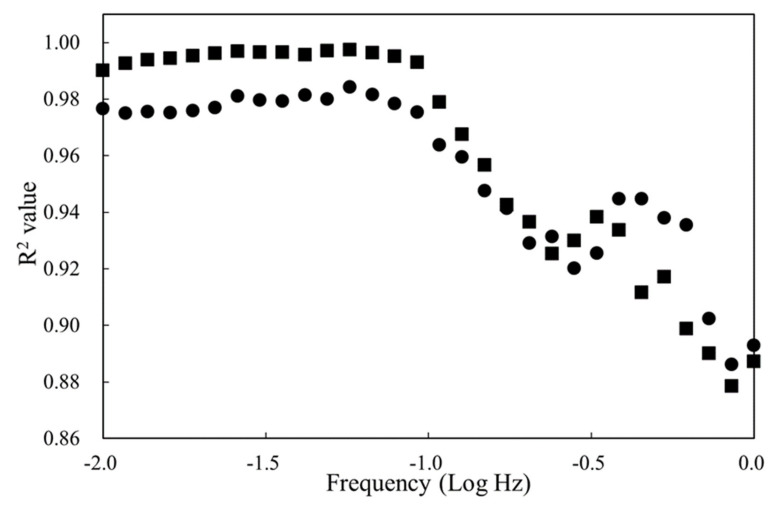
Linear regression results of Log Z at different frequency against AV (■) and TPC (●). *R*^2^: coefficient of determination of the corresponding linear regression.

The linear regression results in Figure 6 also reveal that the Log Z differences at each frequency lower than 0.1 Hz possessed sufficient linearity for accurately quantitating both AV (*R*^2^ > 0.99) and TPC (*R*^2^ > 0.975).

In addition, the relative standard deviations of R_CT_ and Log Z in triplicate tests during the whole deep-frying process were all lower than 7%. These phenomena suggested that both R_CT_ and Log Z could act as reliable quality indicators for determining the discard point of deep-fried oil under repeated usage. While the relatively longer time required to complete a full Nyquist spectrum scan (25 min in this study) is disadvantageous for in-field routine analysis, the Log Z acquired at a given perturbation frequency (the measurement times at 1 Hz and 0.01 Hz were only 3 s and 3 min, respectively) could be an alternative for real-time screening purposes.

In comparison to a similar study [14] using planar-interdigitated electrodes as an impedimetric sensing probe for the quality control of idle heating oil (without frying food), our work not only demonstrated a more simple and economic design of electrochemical cell for evaluating the oil deterioration under a practical frying condition (with French fries), but also successfully achieved good correlations with two regulatory quality indicators, AV and TPC. It may represent a highly anticipated handheld tool for commercial frying operators and official agencies.

Miniaturizing such a high-impedance measurement system (>10 MΩ) with a low-frequency excitation range (<1 Hz) is challenging due to the lack of a dedicated IC chip on the market. A microsystem composed of a dual AD5933 impedance converter configuration and an extended input circuitry has been proven to allow impedance measurement up to 10 GΩ with a frequency scanning bandwidth down to 0.01 Hz [19], which just meets our impedimetric sensing requirements, together with the portability achievement. Although the measured R_CT_ value of fresh palm oil was somewhat higher than 10 GΩ, it still showed high practicability for in-field monitoring of the deterioration of deep-frying palm oil using the proposed simple and economic architecture of an electrochemical cell.

## 4. Conclusions

The constructed EIS-based system provides a simple and economic sensing alternative for label-free and reagent-free determination of the deteriorated condition of deep-frying oil without any need for sample pretreatment procedures. The good correlations of the impedimetric sensing signals (R_CT_ and Log Z) with two recommended quality indicators (AV and TPC) reveal a reliable tool for accurately examining the timing at which the repeated cooking oil should be discarded. By optimizing the geometry of the electrochemical chamber, not only was the reproducibility effectively improved, but the measured impedance could also be reduced to a proper extent for achieving portability by incorporating commercially available IC chips. The realization of a dedicated handheld equipment, together with the production of a corresponding test strip using other carbon paste materials for better sample distribution, is currently under investigation.

## Figures and Tables

**Table 1:** Impedance analysis of fresh palm oil with various electrochemical cell geometries based on the simplified Randles equivalent circuit model without the Warburg diffusion element.

Geometry	Distance (L, cm)	Area (A, cm^2^)	Cell Constant (cm^−1^)	R_S_ (MΩ)	C_D_ (pF)	R_CT_ (GΩ)
#1	0.0385	19.63 × 10^2^	19.61 × 10^−2^	860 ± 303	19.83 ± 2.89	165.33 ± 30.14
#2	0.0275	19.63 × 10^2^	14.01 × 10^−2^	628 ± 177	22.47 ± 2.03	118.50 ± 17.97
#3	0.0165	19.63 × 10^2^	8.40 × 10^−2^	687 ± 338	28.50 ± 1.41	71.83 ± 7.77
#4	0.0055	19.63 × 10^2^	2.80 × 10^−2^	238 ± 179	48.87 ± 3.21	34.97 ± 1.43
#5	0.0055	78.54 × 10^2^	0.70 × 10^−2^	139 ± 80	61.27 ± 2.64	10.67 ± 0.61
#6	0.0055	176.71 × 10^2^	0.31 × 10^−2^	110 ± 46	79.50 ± 2.85	6.17 ± 0.93
#7	0.0055	314.16 × 10^2^	0.18 × 10^−2^	55 ± 17	101.57 ± 5.44	3.95 ± 0.79
#8	0.0055	490.87 × 10^2^	0.11 × 10^−2^	28 ± 16	126.67 ± 2.08	3.21 ± 0.87

The screen-printed carbon electrodes used for geometry #1 to #4 were No. CC-GRP-1 (5 mm in diameter), whereas we used No. CC-GRP-2 for geometry #5 (10 mm in diameter), No. CC-GRP-3 for geometry #6 (15 mm in diameter), No. CC-GRP-4 for geometry #7 (20 mm in diameter), and No. CC-GRP-5 for geometry #8 (25 mm in diameter). This analysis was carried out in triplicate (*n* = 3). All results are presented as the average ± standard deviation.

**Table 2:** Impedance analysis of palm oil during deep-frying process based on the simplified Randles equivalent circuit model without the Warburg diffusion element.

Deep-Frying Time (h)	R_S_ (MΩ)	C_D_ (pF)	R_CT_ (GΩ)
0	291 ± 72	40.47 ± 1.80	10.47 ± 0.49
4	318 ± 92	40.33 ± 1.71	9.81 ± 0.60
8	327 ± 60	41.03 ± 3.40	7.35 ± 0.50
12	249 ± 72	46.03 ± 2.31	6.24 ± 0.36
16	320 ± 92	49.20 ± 7.81	4.31 ± 0.29
20	259 ± 45	47.73 ± 3.60	3.51 ± 0.24
24	353 ± 52	48.70 ± 7.04	2.46 ± 0.17

This analysis was carried out in triplicate (*n* = 3). All results are presented as the average ± standard deviation.

**Table 3:** Viscosity, AV, and TPC during deep-frying process.

Deep-Frying Time (h)	Viscosity (cP)	Acid Value (mg KOH/g Oil)	TPC (%)
0	55.23 ± 0.21	0.21 ± 0.01	4.67 ± 0.29
4	57.90 ± 0.36	0.35 ± 0.01	7.17 ± 0.29
8	60.57 ± 0.15	0.74 ± 0.02	11.17 ± 0.29
12	64.43 ± 0.35	0.90 ± 0.01	16.00 ± 0.50
16	68.47 ± 0.25	1.25 ± 0.03	20.50 ± 0.50
20	73.23 ± 0.25	1.60 ± 0.03	24.17 ± 0.29
24	75.80 ± 0.20	1.91 ± 0.03	26.33 ± 0.29

This analysis was carried out in triplicate (*n* = 3). All results are presented as the average ± standard deviation.

## Data Availability

The data are contained within the article.
